# Evaluation of an automatic image classifier for analysis of bacterial growth on a multiple-agar plate developed for bovine mastitis

**DOI:** 10.1371/journal.pone.0318698

**Published:** 2025-02-20

**Authors:** Lena-Mari Tamminen, Josef Dahlberg

**Affiliations:** Department of Clinical Sciences, Swedish University of Agricultural Science, Uppsala, Sweden; Suez Canal University, EGYPT

## Abstract

Rapid identification of mastitis-causing bacteria is crucial for effective treatment decisions. Several multi-media agar plates have been developed to aid pathogen identification on farms or by veterinarians, but these methods require trained operators. Advances in AI-based automatic image analysis have shown potential for detecting bacterial growth on agar plates in both agriculture and medicine. This study aimed to evaluate the accuracy of an AI-based image bacterial classifier compared to a gold-standard laboratory assessment. A secondary objective was to examine how sample transportation affects diagnoses by comparing results from an on-farm bacterial classifier with those from a laboratory-placed classifier. A total of 1,299 milk samples were collected and analysed at the Swedish Veterinary Agency’s Mastitis Laboratory using both accredited laboratory standards and the bacterial classifier. The image classifier is capable of identifying growth of eight different bacteria types on SELMA + multi-agar plates. Out of 1,212 samples that met the analysis criteria, the bacterial classifier provided diagnoses for 70%, while 30% required further evaluation. The classifier demonstrated high specificity for all diagnoses and high sensitivity for common pathogens such as *Escherichia coli, Staphylococcus aureus*, and non-beta-haemolytic streptococci, though sensitivity was lower for less common pathogens. In a subset of samples analysed by both on-farm and in-lab classifiers and the Mastitis Laboratory, 62% showed consistent diagnoses. The average transportation time was 4.9 days, which influenced bacterial growth. Interestingly, fewer mixed infections were detected post-transport. Automated image classifiers, like Bacticam, hold promise for on-farm mastitis diagnosis, supporting targeted antibiotic treatment and reducing antimicrobial use.

## Introduction

Identification of mastitis causing bacteria is in many aspects crucial, if done rapidly it can be used as a decision tool for selective treatment of clinical cases and even if it is not done rapidly it provides important information about pathogens circulating in the herd. Clinical mastitis is, in Sweden and many other countries, the main reason for antibiotic treatment of dairy cows [1]. Even though antibiotic treatment often is beneficial for a cow suffering from clinical mastitis it is not always justified. The self-cure rate for mastitis caused by *Escherichia coli* is so high that antibiotic treatment rarely is warranted [2]. Evaluation of on-farm mastitis tests has shown that incorporating the results in treatment decision-making for clinical cases, but not sub-clinical cases, resulted in more targeted treatments and less antibiotic use [3]. Further, a recent meta-analysis showed no adverse effects on production and infection related parameters when using selective mastitis treatment (i.e., identifying the pathogen before treatment) over blanket treatment for mild and moderate cases of mastitis [4]. Identification of mastitis causing bacteria is as such an important tool to designate correct treatment and reduce the antimicrobial use in the dairy industry.

**Fig 1:**
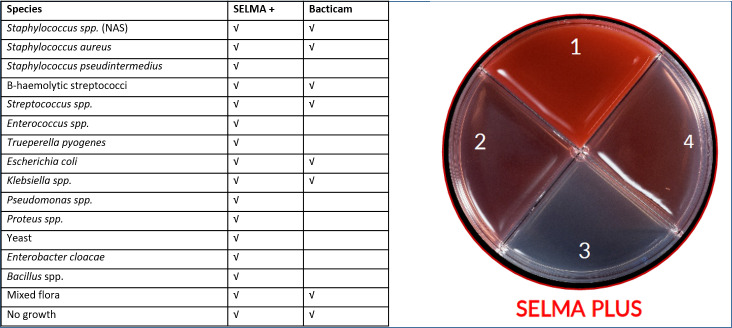
List of growth possible to identify on SELMA + and included agars. Bacterial growth that according to the manufacturer is possible to identify on SELMA + and growth that bacterial classifier (Bacticam) can identify. The agars on the SELMA + are 1) bovine blood with esculine, 2) MacConkey, 3) PGUA and 4) mannitol salt.

The need for rapid and easy identification of mastitis causing pathogens has led to the development of multiple-media agar plates, e.g., Accumast (FERA Animal Health, College Station, TX, USA), SSGN and SSGNC (Eurofins, Luxembourg, Luxembourg), Minnesota Easy Culture Systems (University of Minnesota, St. Paul, MN, USA), *VetoRapid* (Vétoquinol, Bern, Switzerland), SELMA and SELMA PLUS (SELMA + , Swedish Veterinary Agency (SVA), Uppsala, Sweden). SELMA + is a multiple-agar plate with four different agars intended to use for identification of clinical mastitis causing pathogens. It is possible to identify 14 different species or groups of microorganisms as well as mixed flora and no growth on SELMA + (Fig 1). Identification of bacteria/ microorganisms is based on the appearance and growth of the colonies on the different agar fields and in some cases colour and odour [5].

Automatic image analysis has in later years become applicable to a wide range of subjects and fields. In agriculture, automatic image analysis has been described for different areas ranging from growth in broiler chickens to plant recognition, reviewed by Kamilaris et al. [6]. In medicine automatic image analysis are used for Gram-staining and smears, as reported by Smith et al. [7] in a review on infectious disease. In bacteriology automatic image analysis has been used to detect presence or absence of bacterial colonies on agar plates [8,9], and also to detect specific bacterial growth on a chromogenic media [10–12]. In mastitis diagnostics, an AI-based automatic image analysis has been used to identify clinical mastitis-causing pathogens on chromogenic multiple-agar plates [13].

A bacterial classifier, Bacticam, for automatic reading and classification of bacterial growth on SELMA + multiple-agar plates has been developed by the company Agricam (Linköping, Sweden). The bacterial classifier is based on an artificial neural network doing automatic image analysis of the multiple-media agar plates. The bacterial classifier can currently distinguish between eight different types of bacterial growth (including mixed flora and no growth, Fig 1) and the system is developed to be used on farms by trained farm personnel.

The aims of this study were to; 1) evaluate the accuracy of the automatic bacterial classifier compared to a (gold) standard laboratory culture, 2) explore the variation in results after transportation of samples.

## Materials and methods

### Samples and laboratory procedure

Milk samples from dairy cows with clinical mastitis arriving at the Mastitis Laboratory, at the Swedish Veterinary Agency (SVA) in Uppsala, Sweden, were used in this study. SVA is a reference laboratory and the Mastitis Laboratory is accredited according to ISO 17025. At the Mastitis Laboratory milk samples were handled in parallel and analysed with two different methods. The milk samples used in this study came from two different sources, either dairy farms with a Bacticam station in use (Agricam clients) or other farms. Approval to use the milk samples for research purpose was obtained from the sample owner, either directly (by signing a form) or indirectly (by accepting the terms and conditions when sending a sample to SVA). Ethical approval for the study was not warranted according to the Swedish animal welfare ordinance (2019:66). Milk samples arriving at the Mastitis Laboratory from the 8^th^ of July 2021 to the 6^th^ of April 2022 were included in the study. The distribution of milk samples over time and sample origin are presented in Supporting information (S1 Fig).

At the Mastitis Laboratory, milk samples were processed according to NMC recommendations [14]. In brief, 10 µl of milk were cultured aerobically on a blood agar plate at 37° C and evaluated after 24 and 48 hours of growth. At evaluation, number of colonies were registered and initially characterised based on morphology and haemolysis. For bacteriological identification, bacterial colonies from agar plates were typed to species level through MALDI-Tof analysis. If necessary, an extended bacteriological examination was performed, in which case the detection limit was lowered by spreading 100 µl of milk on the agar plate. Bacterial diagnosis at the Mastitis Laboratory is based on colony morphology and a sample is considered positive if at least two CFU of a major pathogen or at least five CFU of a minor pathogen are identified. If growth of two different colony types are detected the sample is classified as having a mixed flora and if three or more different colonies are detected, the sample is considered contaminated. However, if a highly contagious (major) pathogen is identified among numerous different colonies the sample will be classified as positive for both, i.e., co-infected.

In parallel with the accredited method, personnel from the Mastitis Laboratory spread 4 x 10 µl of milk from each sample on a SELMA + agar plate and photographed the growth in a Bacticam photo studio provided by Agricam. The Bacticam photo studio is a light impervious cubical box approximately 20 x 20 x 20 cm large, with a slot for the agar plate, a camera holder and internal illumination. Apart from the photo studio, equipment for growing bacteria and a smartphone camera with Internet access is required for the Bacticam bacterial analysis. After spreading, SELMA + agar plates were incubated aerobically at 37 °C and photos were taken after 24 and 48 hours of growth. Images from the photographed SELMA + agar plates were temporarily stored in a OneDrive folder before being downloaded to a SLU server to which only the authors had access.

Images of the photographed SELMA + agar plates were analysed in an off-line image classifier system that was provided by Agricam to the authors. The image classifier system is a two-step process where images first are quality controlled and then classified by a pre-trained image classifier algorithm. The demands for the off-line image classifier algorithm were that 4 images were available, these 4 images needed to be taken with illumination of the agar plate from above and below with a minimum of 18 hours apart. Samples with less than 4 images available were excluded. Samples with more than 4 images available were manually curated so that surplus images were excluded from the analysis. The bacterial classifier can only identify one bacterial diagnosis per sample. For the commercial on-line Bacticam system, the company have routines for correcting surplus or missing images, the option of a preliminary bacteriological diagnosis after 8–24 hours as well as a backup system with manual inspection or laboratory analysis for un-diagnosed samples.

Results from the (gold) standard laboratory culture and the off-line image classifier are the primary source of data for this study. A third source of data was provided by the company Agricam and consisted of image classifier results from on-farm Bacticam stations from milk samples that were also sent to the Mastitis Laboratory during the period 8^th^ of July to 29^th^ of December 2021.

### Data handling and statistical analysis

Raw data was compiled using Microsoft Excel and data analysis was performed in the statistics program R [15]. Analysis was performed on three levels.

First, an overview of how the classifier performs in practice was performed (general performance). This includes descriptive presentation (e.g., to which extent bacterial diagnoses are provided), exploration of patterns for when a diagnosis was not provided and how the classifier performed for samples with co-infection. Identified bacterial species were grouped to the lowest comparable taxonomic level. Bacteriological species (/genera) for which the classifier had no pre-training were categorized as “Other” and the classifier was expected to classify them as “Additional evaluation required”. These patterns were explored descriptively.

In the second level, diagnoses provided by the classifier were compared to diagnoses from the reference (gold standard) laboratory to calculate sensitivity, specificity and predictive values (specific performance). In this comparison only samples for which the bacterial classifier returned a diagnosis and where the reference laboratory specified one bacterial diagnosis per sample were included (i.e., samples with co-infection and samples with a specified species in mixed flora were excluded). Identified bacterial species were grouped to the lowest comparable taxonomic level. For calculation of sensitivity, specificity and predictive values the package DTCompair was used [16] and for visualization the package ggplot2 was used [17].

The third level of the analysis includes comparisons of results from the on-line classifier from on-farm Bacticam stations (third source of data) in relation to the results of the off-line classifier at the reference laboratory and the reference laboratory results. Descriptively exploring overall accordance, the effect of sample transportation and sample handling.

## Results

### General performance

During the study period, 1299 milk samples were analysed by the Mastitis Laboratory, the majority of the samples arrived between September and December, 52% (672/1299) of the milk samples came from farms with a Bacticam station in use and 48% of the samples came from other farms (Supl. S1 Fig ). All samples were cultured and photographed in the photo studio. Images from 87 samples could not be analysed by the classifier due to poor image quality or fewer than 4 images per sample and were excluded from the study. Of the remaining 1212 samples, 1077 samples had one specific bacterial diagnose identified per sample and 135 samples had multiple pathogens identified per sample. Extended bacteriological examination was performed by the Mastitis Laboratory on 110 samples. The classifier returned bacterial diagnoses for 845 samples (70%) while 367 (30%) required additional evaluation. A summary of the classifiers’ response in relation to the Mastitis Laboratory diagnosis is presented in Table 1 and as a more detailed list in Supporting information (S1 Table).

**Table 1:** Summary of bacterial classifiers response in relation to Mastitis Laboratory bacterial diagnosis.

		Single bacterial diagnose				Multiple bacterial diagnoses (not trained for)	
	Total	Specific pathogen (trained for)	Mixed flora (trained for)	No growth (trained for)	Other species (Not trained for)	Specific growth + mixed flora	Co-infection + mixed flora
Additional evaluation	367 (30%)	188 (16%)	43 (4%)	30 (2%)	49 (4%)	20 (2%)	37 (3%)
Bacterial diagnosis provided	845 (70%)	541 (45%)	99 (8%)	66 (5%)	61 (5%)	36 (3%)	42 (3%)

Of the 367 samples that according to the classifier required additional evaluation, 261 samples had bacterial growth that the image classifier theoretically could identify. 57 samples had growth of multiple bacterial species in the same sample and 49 samples had growth of a single bacterial species for which the classifier had not been trained, i.e., for 106 samples the correct Bacticam diagnosis would be “additional evaluation required” (Table 1). Further, 32 out of the 367 samples had been subjected to extended bacteriological examination, the majority (27/32) of these samples were classified as “no growth” by the Mastitis Laboratory, in the remaining five samples, either *E. coli* or *Staphylococci* in mixed flora were identified.

**Fig 2:**
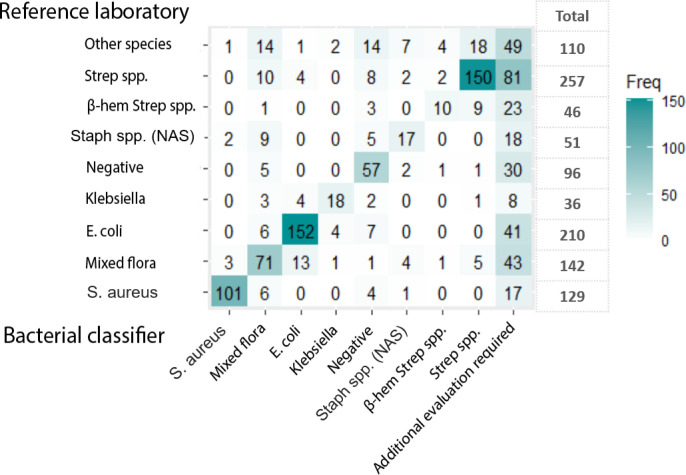
Comparison of bacteriological diagnoses reported by the reference laboratory and the bacterial classifier. 1077 samples collected from dairy cows with clinical mastitis, analysed with both methods and with a single bacterial diagnose per sample*.*

A comparison of Mastitis Laboratory and classifier results for samples with one bacterial diagnosis per sample is presented in Fig 2. The most frequently occurring bacterial diagnoses were *E. coli, Streptococcus spp.,* mixed flora and *S. aureus*. The fifth most common bacterial diagnosis was “other species”, i.e., species for which the classifier had not been trained.

In total, 110 samples had, according to the Mastitis Laboratory, growth of species for which the classifier had not been trained. Forty-nine of these 110 samples received a correct “additional evaluation required” diagnosis by the classifier while an incorrect bacterial diagnosis was returned for 61 samples. The most commonly returned diagnosis in these cases was *Strep.* spp. (n = 18), mixed flora (n = 14) and negative growth (n = 14) (Fig 2).

### Specific performance

**Fig 3:**
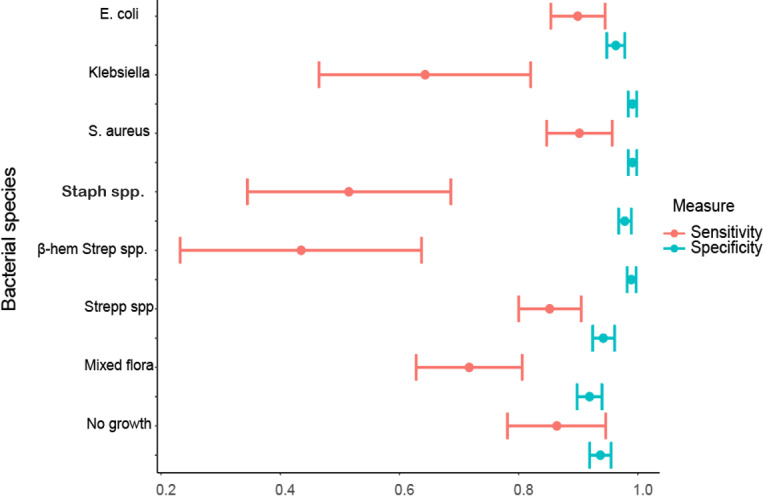
Sensitivity and specificity of bacterial diagnosis provided by the bacterial classifier. Results from a reference laboratory were used as a gold standard. Mean values estimations are presented as centroid with 95% confidence intervals presented as bars.

For calculation of sensitivity, specificity and predictive values, 767 samples for which the Mastitis Laboratory had provided one single bacterial diagnosis per sample and the bacterial classifier had provided a bacterial diagnosis were used (Table 2). The most frequently occurring pathogens according to the Mastitis Laboratory were *E. coli* (n = 169), *S. aureus* (n = 112) and non-beta-haemolytic streptococci (n = 176). Samples with no growth and samples with mixed flora were also relatively common (n = 66 and n = 99) while NAS, *Klebsiella* and beta haemolytic streptococci were less common (n < 35). The sensitivity and specificity of the bacterial classifier varied between bacterial species, with the best performance for the more frequently occurring pathogens (Fig 3). The specificity of the bacterial classifier was high for all bacterial diagnoses with a range from 91.9% to 99.1%. For the sensitivity there was a larger variation and the confidence intervals were broader. The most frequently occurring pathogens (*E. coli*, *S. aureus* and non-beta haemolytic streptococci) had relatively high sensitivity (> 85%) while the sensitivity for the less common pathogens (*Klebsiella spp.*, NAS and β-haemolytic streptococci was lower (43.5%–64.3%) (Fig 3). A table with estimated mean values for sensitivity, specificity, PPV and NPV together with standard error and confidence interval is presented in Supporting information (S2 Table).

**Table 2:** 2 x 2 comparison of bacterial diagnoses. Bacterial diagnoses were identified by Mastitis Laboratory (ML) and the bacterial classifier (classifier) in 767 milk samples.

*E. coli*	ML +	ML−		*Klebsiella spp.*	ML +	ML−
Classifier +	152	22		Classifier +	18	7
Classifier−	17	576		Classifier−	10	732
*S. aureus*	ML +	ML−		Staph. spp (NAS)	ML +	ML−
Classifier +	101	6		Classifier +	17	16
Classifier−	11	649		Classifier−	16	718
beta-hemolytic strep	ML +	ML−		Strep. spp (other)	ML +	ML−
Classifier +	10	8		Classifier +	150	34
Classifier−	13	736		Classifier−	26	557
No growth	ML +	ML−		Mixed flora	ML +	ML−
Classifier +	57	44		Classifier +	71	54
Classifier−	9	657		Classifier−	28	614

### Handling and transport

**Fig 4:**
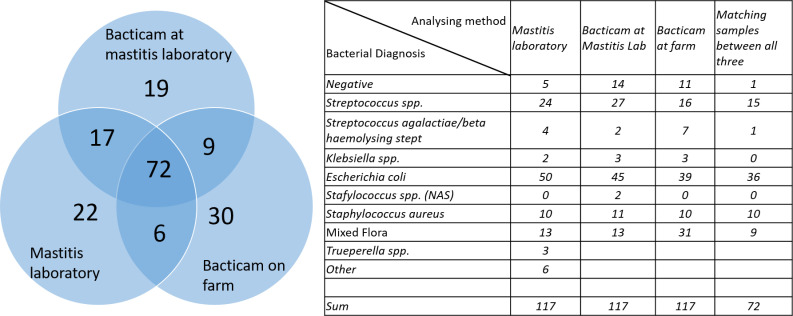
Comparison of results from Mastitis Laboratory and bacterial classifier. The bacterial classifier (Bacticam) were placed either on farm or at Mastitis Laboratory. Only samples with one bacterial diagnosis per sample included. A) Venn diagram showing overlap between analysing method and shared bacterial diagnoses. B) Detailed table with bacterial diagnosis per analysing method.

To evaluate the effect of handling and transportation, bacterial classifier results from the on-line on-farm Bacticam stations provided by Agricam were used. 130 milk samples had comparable results from the Mastitis Laboratory, the off-line bacterial classifier and the on-line bacterial classifier. For these 130 samples, 144 bacterial diagnoses were provided by the Mastitis Laboratory, 117 milk samples that had one bacterial diagnosis per sample were further explored. 72 (62%) of the 117 samples had the same bacteriological diagnosis given by the on-line classifier, the off-line classifier and the reference laboratory, and 69% of the samples (81/117) had the same bacteriological diagnosis given by the on-line classifier and the off-line bacterial classifier. There was a higher concordance of results between the Mastitis Laboratory and the off-line bacterial classifier (Bacticam at laboratory, 89 shared diagnoses) than between the Mastitis Laboratory and the on-line classifier (Bacticam on farm, 78 shared diagnoses). A presentation of the 117 samples that had one bacterial diagnosis per sample and comparable results from the on-line classifier, the off-line bacterial classifier and the Mastitis Laboratory is presented in Fig 4. An interesting observation is that 26% of the samples that were cultured on farm and evaluated by the on-line image classifier had growth of mixed flora, when the same samples were cultured at the reference laboratory and evaluated by the off-line bacterial classifier, 11% of the samples had growth of mixed flora. As the sample results provided by Agricam contained detailed information on when samples were collected, the time from sampling to arrival at the reference laboratory was calculated. The mean transportation time for the milk sample was 4.9 days with a range from 1 – 26 days.

## Discussion

Different methods have been developed for rapid on-farm identification of mastitis causing bacteria with variations in number of bacterial species possible to identify and time to result. When evaluated, the on-farm systems generally perform well regardless if they are compared to farm antibiotic use [3] or compared to a mastitis laboratory result [18,19]. However, it has been shown that trained specialists perform better than untrained observers [20]. Consequently, a problem with on-farm mastitis identification systems is that accurate interpretation of the bacterial growth requires trained and experienced personnel.

Correctly identifying mastitis causing pathogens can be challenging and inter-laboratory variation has been shown to be substantial. A Finish study of inter-laboratory accordance of diagnosis of lyophilized common mastitis pathogens showed an overall agreement of 63-93%, depending on testing round, but also a variation between pathogens where some pathogens (*E. coli, S. aureus)* were always correctly identified while others not [21]. Later studies have shown similar results, of the 381 mastitic milk samples that were sent to two different mastitis laboratories in the study by Ferreira et al. [19], 212 (55%) received the same bacterial diagnosis.

The possibility of using an automated image classifier for bacterial diagnosis will limit the inter-laboratory and inter-personnel variation of interpreting results. In this study, the bacterial classifier performed well for commonly occurring species.

The bacterial distribution in the present study differs from the distribution of clinical mastitis causing bacteria in Sweden. According to a study from Duse et al. [22], the five most common clinical mastitis causing bacteria in Sweden are *S. aureus* (27.8%), *Str. dysgalactiae* (15.8%), *E. coli* (15.1%), *Str. uberis* (11.4%), *T. pyogenes* (7.7%), contaminated samples (i.e., mixed flora) was together with no growth the sixth most common diagnosis (4.9% each). In the present study streptococci were separated based on their haemolytic properties, rather than species level for comparison of results and non-haemolytic streptococci was the most abundant bacteria, followed by *E. coli*, mixed flora, *S. aureus* and other. The group “other” consisted of a wide range of bacteria that the bacterial classifier is not trained to recognise, including *T. pyogenes*. Even though the distribution differ, the subset of samples used in this study is, at large, representative for the mastitis pathogens common in Sweden, the differences that occur can be attributed to selection bias as samples in this study came from a limited number of farms and was collected over a limited time. A list of all the bacterial diagnoses provided by the Mastitis Laboratory and the bacterial classifiers as well as sample origin is provided in Supporting information (S1 Table). The level of co-infected samples in the present study was 11% and is in a similar range as previously reported (9%) from the same laboratory [22,23]. The bacterial classifier has only been trained to identify growth of one pathogen per plate, if several colonies of different types are present, the sample will be classified as having a mixed infection.

### Specific performance

Sensitivity and specificity were evaluated on samples where the Mastitis Laboratory and the bacterial classifier had provided a single bacterial diagnosis per sample. The specificity was overall high for all bacterial diagnoses provided by the classifier, implying that samples without a specified diagnosis really are free from it. A high specificity is important to assure that animals don’t receive unnecessary treatments. For the sensitivity there was a larger variation in results. For the most common bacterial diagnoses in this data set (*E. coli, S. aureus,* non-haemolytic streptococci and no growth) the sensitivity was high, indicating that if the classifier give this response it is most likely correct. For less common bacterial diagnoses (*Klebsiella spp.,* NAS, beta-haemolytic streptococci and mixed flora) the sensitivity was lower and the results less trustworthy. The distribution of bacterial diagnoses in the data set that was provided as training data for the neural network is unknown to us authors, it is not unlikely that the training data had a similar distribution of diagnoses as presented here and that the classifier therefore is better trained on certain diagnoses. For example, *E. coli* and *Klebsiella spp.* have similar but distinct growth patterns on a SELMA + agar plate, with a colour change in the PGUA agar as differentiating factor, this colour change is visually apparent and we do find it probable that the colour change would be possible to identify for an automated image classifier, consequently we find it likely that the sensitivity for *Klebsiella spp.* will increase with more training. The specificity values reported for the Bacticam bacterial classifier in this study (91.9-99.1%) are in line with specificity values reported for a similar application, “Rumi” (96-99%), by Garcia et al. [13]. A variation in sensitivity across different bacterial diagnoses that is noted here was likewise noted by Garcia et al.

### Handling and transport

It is well known that storage can affect the hygienic properties and bacterial content of raw milk. According to literature, total bacterial count will increase with prolonged storage, especially if milk is stored over 4 ^°^C [24]. For microorganisms typically found in milk, it has been shown that there is a large variability in growth rate, both within and between different organisms [25]. This difference in growth rate would affect the original composition of bacteria in a milk sample, and the difference would increase with time. This has been shown in a master degree thesis that concluded that mixed bacterial flora increased with increased number of days of transportation [26]. In the present study, the average transportation time was 4.9 days, the conditions during transport are unknown but milk samples sent to the Mastitis Laboratory are most often transported at ambient temperature (personal message). Considering the wide range in transportation time (1 - 26 days), we cannot exclude that some samples were stored before transportation. Regardless of transportation temperature, the time spent for transportation to the laboratory prevent the analysis from being used as a decision tool for selective treatment. Considering the transportation time, we did expect that there would be more samples with mixed bacterial growth after transportation compared to before, surprisingly the results were opposite (26% mixed infection before transport vs. 11% after). We can only speculate on why this difference occurred, on the farm either the farmer or a trained employee would be responsible for spreading the milk on the agar while in the lab a professional lab-assistant did it. It is possible that the difference in “professionalism” contributed to the difference in results regarding mixed infection and concordance between analysing methods. However, it cannot be ruled out that handling and storage of agar plates or other factors contributed.

## Conclusion

We conclude that the AI-based image analysis bacterial classifier, Bacticam, is showing promise in accurately diagnosing common mastitis pathogens. The classifier performs well with high specificity for all diagnosed pathogens, assuring avoidance of unnecessary treatment. For bacterial diagnoses of *E.coli, S. aureus* and non-haemolytic streptococci the sensitivity of the bacterial classifier is high. An apparent benefit of the bacterial classifier is the reduced time from sampling to results, although operators must take appropriate measures before plating the milk to minimize growth of mixed infection.

## Supporting information

S1 Fig Number of samples included in the study presented as total samples per month and separated based on origin. (PDF)

S1 Table Bacterial diagnosis provided by bacterial classifier (Bacticam) and Mastitis Laboratory (ML) and sample origin for all samples included in the study. (PDF)

S2 Table Statistical accuracy of diagnoses provided by bacterial classifier (Bacticam) for 767 milk samples from cows with clinical mastitis. (PDF)

S1 File Anonymized bacteriological diagnoses from the three sources used in this study. (CSV)
