# Supplementary material for: Evaluation of an automatic image classifier for analysis of bacterial growth on a multiple-agar plate developed for bovine mastitis
Source: PLoS One. 2025 Feb 20;20(2):e0318698. doi: 10.1371/journal.pone.0318698 (PMC11841905; doi:10.1371/journal.pone.0318698)
Supplement: S1 Fig — (PDF) [file pone.0318698.s001.pdf]

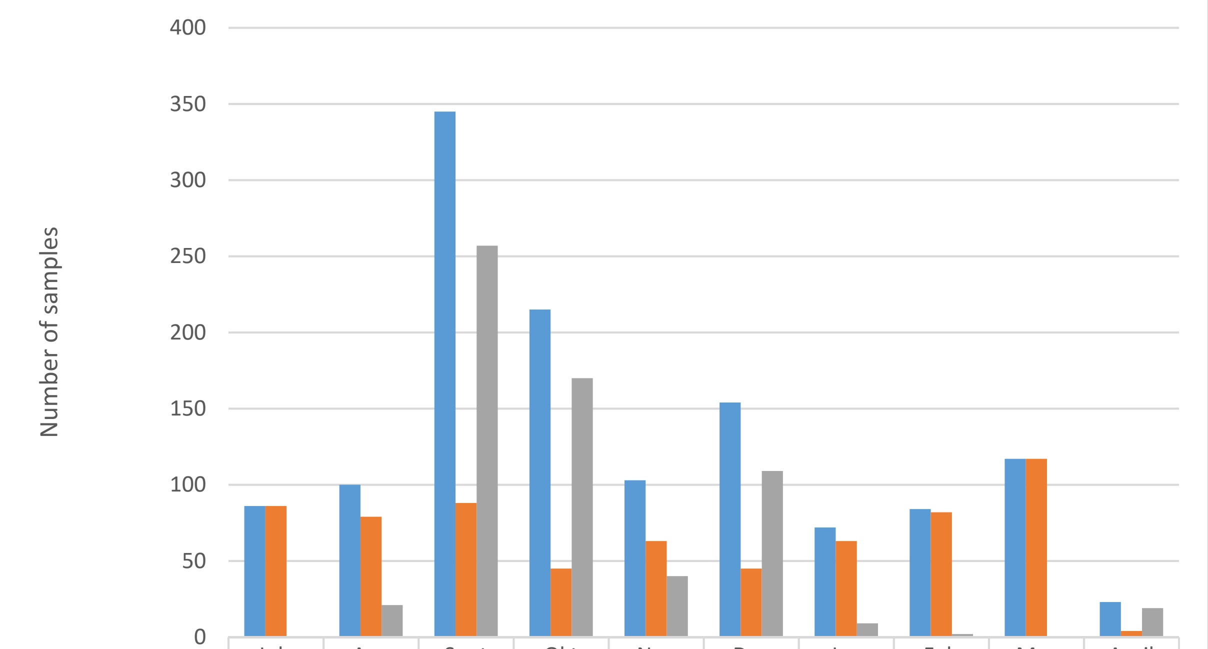

	July	Aug	Sept	Okt	Nov	Dec	Jan	Feb	Mar	April
All	86	100	345	215	103	154	72	84	117	23
Bacticam farms	86	79	88	45	63	45	63	82	117	4
Other farms		21	257	170	40	109	9	2		19

S1 figure. Number of samples included in the study presented as total samples per month and separated based on origin.
