# Supplementary material for: Evaluation of an automatic image classifier for analysis of bacterial growth on a multiple-agar plate developed for bovine mastitis
Source: PLoS One. 2025 Feb 20;20(2):e0318698. doi: 10.1371/journal.pone.0318698 (PMC11841905; doi:10.1371/journal.pone.0318698)
Supplement: S1 Table — (PDF) [file pone.0318698.s002.pdf]

S1 Table. Bacterial diagnosis provided by bacterial classifier (Bacticam) and Mastitis Laboratory (ML) and sample origin for all samples included in the study.

		Analyzed by		Sample origin	
Bacteriological diagnosis (grouped for comparison)		Bacticam	ML	Other farm	Bacticam farm
Escherichia coli		182	239	92	147
Staphylococcus aureus		112	177	84	93
Beta-haemolytic streptococci/ *Str. agalactiae*		24	64	24	40
Klebsiella spp.		28	47[1]	25	22
Mixed Flora		155	219[5]	106	113
Staphylococci, other (NAS)		35	51[2]	26	25
Streptococcus spp.		205	353[3]	184	169
Trueperella spp.			109	55	54
Negative / No growth		104	101	46	55
Other			91[4]	49	42
Additional Evaluation Required		367			
Poor Images		87			
Total		1299	1451	691	760

[1] *Klebsiella oxytoca, K. pneumoniae,* K. species

[2] *Staphylococcus chromogenes, S. epidermidis, S. haemolyticus, S. hyicus, S.sciuri, S. simulans, S. xylosus*. All either penicillinaze positive or negative

[3] *Streptococcus canis* (n=2), *S. dysgalactiae, S. gallolyticus, S. parauberis, S. uberis*

[4] Acinetobacter spp., beta-haemolytic streptococci (n=1), Citrobacter spp., *Corynebacterium bovis*/spp., Enterobacterum spp., Enterococcus spp., *Helcococcus ovis*/spp., *Lactococcus lactis*/spp., Pantoea spp., *Proteus mirabilis*/spp., *Pseudomonas aerginosa/fluorescens*, Serratia spp., Trichosporon spp., Yeast

[5] Mixed flora as single diagnosis for 154 samples
