# Supplementary material for: Evaluation of an automatic image classifier for analysis of bacterial growth on a multiple-agar plate developed for bovine mastitis
Source: PLoS One. 2025 Feb 20;20(2):e0318698. doi: 10.1371/journal.pone.0318698 (PMC11841905; doi:10.1371/journal.pone.0318698)
Supplement: S2 Table — (PDF) [file pone.0318698.s003.pdf]

S2 Table. Statistical accuracy of diagnoses provided by bacterial classifier (Bacticam) for 767 milk samples from cows with clinical mastitis.

		Estimate	SE	CI lower	CI upper
E. coli					
	Sensitivity	0.899	0.023	0.854	0.945
	Specificity	0.963	0.008	0.948	0.978
	PPV	0.873	0.025	0.824	0.923
	NPV	0.971	0.007	0.958	0.985
Klebsiella spp.					
	Sensitivity	0.643	0.091	0.465	0.820
	Specificity	0.991	0.004	0.984	0.998
	PPV	0.720	0.090	0.544	0.896
	NPV	0.987	0.004	0.978	0.995
S. aureus					
	Sensitivity	0.902	0.028	0.847	0.957
	Specificity	0.991	0.004	0.984	0.998
	PPV	0.944	0.022	0.900	0.988
	NPV	0.983	0.005	0.974	0.993
Staff other (NAS)					
	Sensitivity	0.515	0.087	0.345	0.686
	Specificity	0.978	0.005	0.968	0.989
	PPV	0.515	0.087	0.345	0.686
	NPV	0.978	0.005	0.968	0.989
Beta haemolytic strept.					
	Sensitivity	0.435	0.103	0.232	0.637
	Specificity	0.989	0.004	0.982	0.997
	PPV	0.556	0.117	0.326	0.785
	NPV	0.983	0.005	0.973	0.992
Strepp. Spp.					
	Sensitivity	0.852	0.027	0.800	0.905
	Specificity	0.942	0.010	0.924	0.961
	PPV	0.815	0.029	0.759	0.871
	NPV	0.955	0.009	0.939	0.972
No growth					
	Sensitivity	0.864	0.042	0.781	0.946
	Specificity	0.937	0.009	0.919	0.955
	PPV	0.564	0.049	0.468	0.661
	NPV	0.986	0.004	0.978	0.995
Mixed flora					
	Sensitivity	0.717	0.045	0.628	0.806
	Specificity	0.919	0.011	0.898	0.940
	PPV	0.568	0.044	0.481	0.655
	NPV	0.956	0.008	0.941	0.972

SE; standard error, PPV; positive predictive value, NPV; negative predictive value, Cl lower and Cl upper is the lower and upper values for the 95% confidence interval.
